# Protective Effects of Tormentic Acid, a Major Component of Suspension Cultures of *Eriobotrya japonica* Cells, on Acetaminophen-Induced Hepatotoxicity in Mice

**DOI:** 10.3390/molecules22050830

**Published:** 2017-05-18

**Authors:** Wen-Ping Jiang, Shyh-Shyun Huang, Yoshikazu Matsuda, Hiroshi Saito, Naoto Uramaru, Hui-Ya Ho, Jin-Bin Wu, Guan-Jhong Huang

**Affiliations:** 1 School of Pharmacy, China Medical University, No. 91, Hsueh-Shih R., Taichung 40402, Taiwan; u101053651@cmu.edu.tw (W.-P.J.); sshuang@mail.cmu.edu.tw (S.-S.H.); 2 Nihon Pharmaceutical University, 10281, Komuro, Ina-machi, Kitaadachi-gun, Saitama 3620806, Japan; yomatsuda@nichiyaku.ac.jp (Y.M.); hiroshis@nichiyaku.ac.jp (H.S.); uramaru@nichiyaku.ac.jp (N.U.); 3 Jen Li Biotech Company Ltd., Taiping District, Taichung 41143, Taiwan; jlbioaya@gmail.com; 4 Department of Chinese Pharmaceutical Sciences and Chinese Medicine Resources, China Medical University, Taichung 404, Taiwan

**Keywords:** tormentic acid, acetaminophen, hepatoprotective, HO-1 (heme oxygenase-1), mapk, NF-κB, antioxidation, anti-inflammation, reactive oxygen species.

## Abstract

An acetaminophen (APAP) overdose can cause hepatotoxicity and lead to fatal liver damage. The hepatoprotective effects of tormentic acid (TA) on acetaminophen (APAP)-induced liver damage were investigated in mice. TA was intraperitoneally (i.p.) administered for six days prior to APAP administration. Pretreatment with TA prevented the elevation of serum aspartate aminotransferase (AST), alanine aminotransferase (ALT), total bilirubin (T-Bil), total cholesterol (TC), triacylglycerol (TG), and liver lipid peroxide levels in APAP-treated mice and markedly reduced APAP-induced histological alterations in liver tissues. Additionally, TA attenuated the APAP-induced production of nitric oxide (NO), reactive oxygen species (ROS), tumor necrosis factor-alpha (TNF-α), interleukin-1beta (IL-1β), and IL-6. Furthermore, the Western blot analysis showed that TA blocked the protein expression of inducible NO synthase (iNOS) and cyclooxygenase-2 (COX-2), as well as the inhibition of nuclear factor-kappa B (NF-κB) and mitogen-activated protein kinases (MAPKs) activation in APAP-injured liver tissues. TA also retained the superoxidase dismutase (SOD), glutathione peroxidase (GPx), and catalase (CAT) in the liver. These results suggest that the hepatoprotective effects of TA may be related to its anti-inflammatory effect by decreasing thiobarbituric acid reactive substances (TBARS), iNOS, COX-2, TNF-α, IL-1β, and IL-6, and inhibiting NF-κB and MAPK activation. Antioxidative properties were also observed, as shown by heme oxygenase-1 (HO-1) induction in the liver, and decreases in lipid peroxides and ROS. Therefore, TA may be a potential therapeutic candidate for the prevention of APAP-induced liver injury by inhibiting oxidative stress and inflammation.

## 1. Introduction

Acetaminophen (APAP) is considered a safe and effective drug at therapeutic dosages and is commonly used as an antipyretic and analgesic agent [1]. However, an APAP overdose causes severe liver damage that has the potential to progress to liver failure, which accounts for nearly half of the liver failure cases in the USA and a significant portion of cases in Europe [2]. In Taiwan, an APAP overdose is also an important cause of acute liver failure [3]. An APAP toxic metabolite, *N*-acetyl-*p*-benzoquinone imine (NAPQI), is believed to be responsible for APAP-induced hepatotoxicity. Following an APAP overdose, high levels of APAP toxic metabolites deplete the hepatic endogenous antioxidant glutathione (GSH) pool and cause oxidative stress [4], further inducing mitochondrial toxicity and ultimately leading to cell death [5]. A large body of evidence indicated that oxidative stress is involved in acetaminophen toxicity [6,7].

Inflammatory responses may be involved in the APAP-induced pathophysiology. Hepatic macrophages were proposed to contribute to APAP-induced liver injury through the production of pro-inflammatory cytokines and mediators, such as tumor necrosis factor-alpha (TNF-α), interleukin-1 beta (IL-1β) [8], and IL-6 [9]. These observations revealed an association between APAP-induced liver injury and several inflammatory mediators, such as certain cytokines that modify the toxicity of APAP [10].

*Eriobotrya japonica* Lindl. (Loquat) is an evergreen fruit tree in the Rosaceae family, and leaves of the plant have been used in traditional medicine for the treatment of cough, chronic bronchitis, asthma, inflammatory diseases, diabetes, and cancer [11,12,13,14]. Triterpenes from the extract of loquat leaf exert various pharmaceutical effects [13,14], including anti-inflammatory [15,16,17], antioxidative [18], and hepatoprotective effects [19], and large amounts of triterpenes can be obtained from callus tissue cultures of loquat leaf [20,21]. 

**Figure 1:**
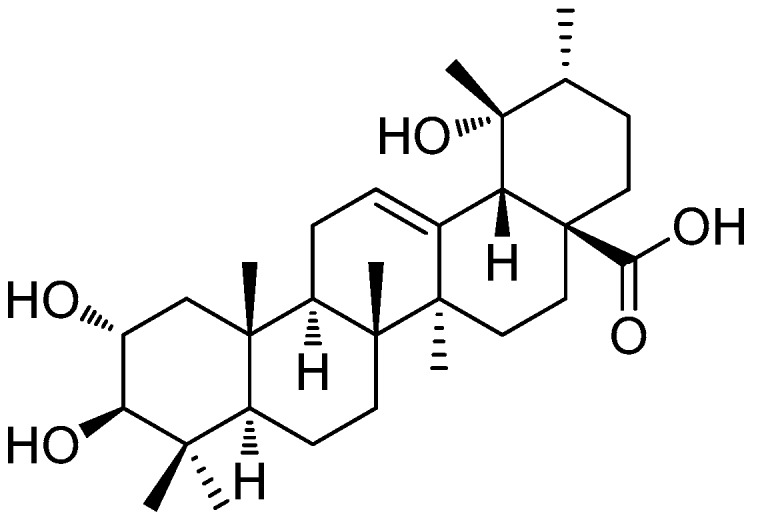
Structure of tormentic acid.

In this context, we highlighted tormentic acid (TA) (Figure 1), one of the main pentacyclic triterpenes isolated from the bioreactor-cultured suspension callus of *Eriobotrya japonica* [22]. TA has been reported to exhibit anticancer [23,24,25], antibacterial [26], anti-inflammatory [27,28,29], and anti-atherogenic properties [30,31]. TA was also suggested to be a potential treatment for type 2 diabetes and hyperlipidemia [32], and has a potential hepatoprotective effect [27,33]. However, little information is available about the hepatoprotective effects of TA on APAP-induced liver injury. Thus, we investigated the effects of different doses of TA by comparing them to *N*-acetylcysteine (NAC), a clinical treatment for APAP overdose [21], in an animal model of acute APAP-induced liver injury.

## 2. Results

### 2.1. Effect of TA on Hepatotoxicity in APAP-Treated Mice

**Figure 2:**
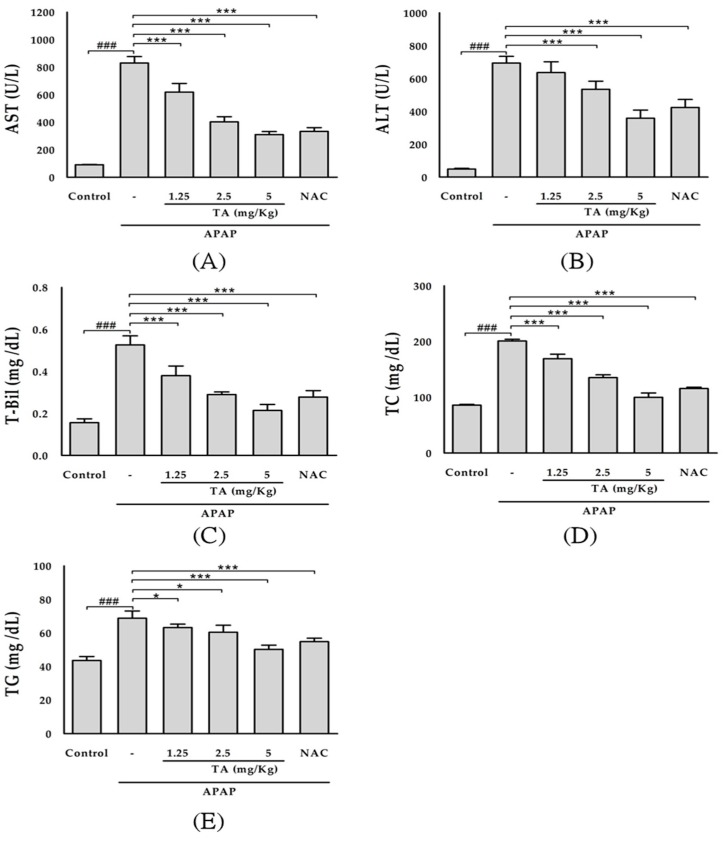
Effects of tormentic acid on serum AST (**A**); ALT (**B**); T-Bil (**C**); TC (**D**); and TG (**E**) in APAP-induced mice. The values are reported as the means ± S.E.M. of five mice per group. ^###^ *p* < 0.01 compared with the control group; * *p* < 0.05, and *** *p* < 0.001 compared with the APAP group.

The serum levels of several hepatic enzymes, such as AST, ALT, and T-Bil, were used as biochemical markers of early acute hepatic damage. The serum levels of AST, ALT, and T-Bil were measured to evaluate hepatic tissue damage (Figure 2A–C). APAP administration resulted in a significant (*p* < 0.001) increase in the AST, ALT, and T-Bil levels compared with those in the control group. The intraperitoneal pre-administration of three different doses of TA (1.25, 2.5, and 5 mg/kg) significantly prevented the increase in the serum ALT and AST levels. Treatment with 600 mg/kg NAC (positive control) also prevented the increase in the AST, ALT, and T-Bil levels. Moreover, APAP administration increased the serum TC and TG levels (Figure 2D,E). The intraperitoneal pre-administration of three different doses of TA (1.25, 2.5, and 5 mg/kg) significantly prevented the increase in the serum TG and TC levels. The administration of 600 mg/kg NAC (positive control) also prevented the increase in the TG and TC levels.

### 2.2. Histopathology of the Liver

**Figure 3:**
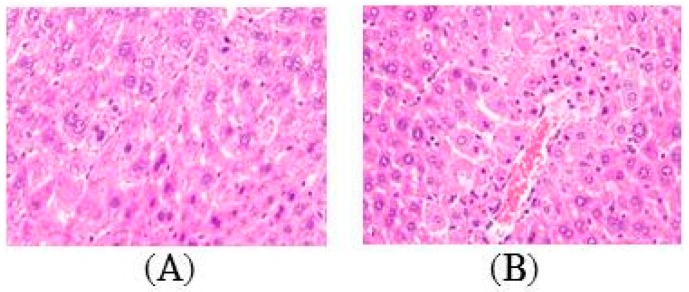

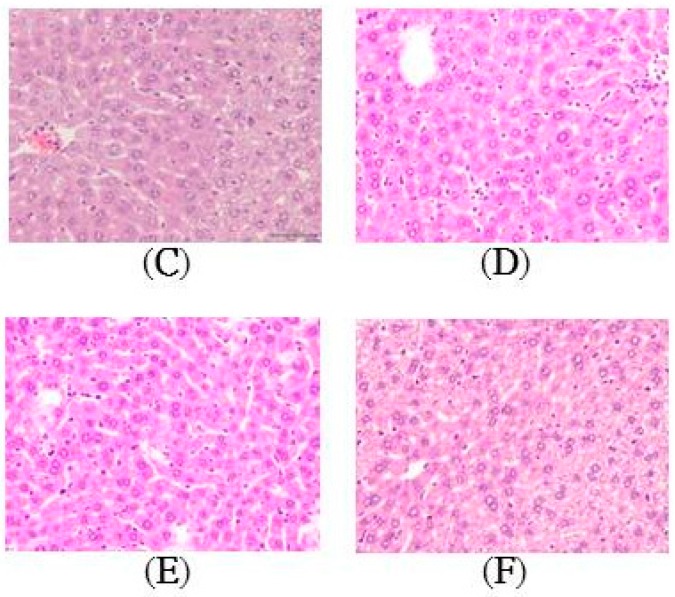
Effects of tormentic acid on APAP-induced liver damage. Sections were stained with H & E (400×) and observed under a light microscope. Control (**A**); APAP (400 mg/kg) (**B**); TA (1.25 mg/kg) + APAP (400 mg/kg) (**C**); TA (2.5 mg/kg) + APAP (400 mg/kg) (**D**); TA (5 mg/kg) + APAP (400 mg/kg) (**E**); NAC (600 mg/kg) + APAP (400 mg/kg) (**F**).

Based on the analysis of the hematoxylin and eosin (H & E)-stained tissue section, APAP intoxication generated extensive changes in liver morphology, including steatosis, inflammation, hepatocyte ballooning, and necrosis (Figure 3B). TA pretreatment apparently alleviated liver damage, as shown by the decreased necrotic areas and hepatocellular degeneration (Figure 3C–E). This finding was consistent with the levels of the enzyme markers.

### 2.3. Effect of TA on Lipid Peroxidation in APAP-Treated Mouse Livers

**Figure 4:**
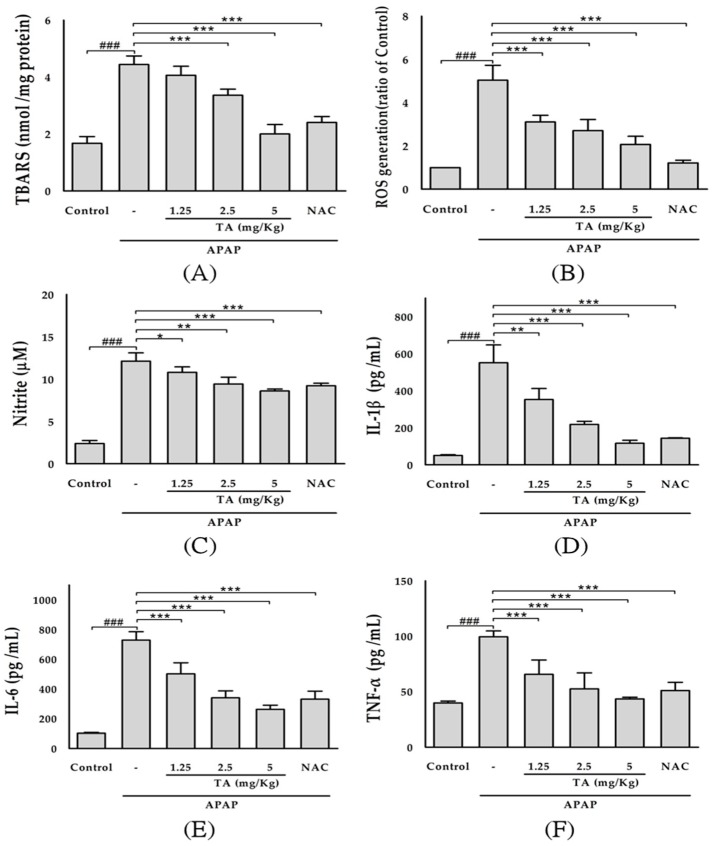
Effects of TA on liver lipid peroxides levels (**A**); Effects of TA on serum ROS (**B**); NO (**C**); IL-1β (**D**); IL-6 (**E**); and TNF-α levels (**F**) in APAP-induced mice. The values are reported as the means ± S.E.M. of five mice per group. ^###^ *p* < 0.01 compared with the control group; * *p* < 0.05, ** *p* < 0.01, and *** *p* < 0.001 compared with the APAP group.

As shown in Figure 4A, the hepatic levels of thiobarbituric acid reactive substances (TBARS) were assessed as an indicator of lipid peroxidation in the tissue. Mice treated with APAP alone exhibited a significant increase (*p* < 0.001) in the tissue TBARS levels. TA (1.25, 2.5, and 5 mg/kg) notably prevented this increase in the TBARS levels compared to those in the APAP group. Moreover, NAC also prevented the elevation of the TBARS levels in the liver and maintained them at normal values.

### 2.4. Effect of TA on Serum ROS Levels in APAP-Treated Mice

As shown in Figure 4B, the serum ROS levels were significantly increased in APAP-treated mice compared to those in the control group (*p* < 0.001). TA pretreatment significantly reduced ROS production in the APAP-treated mice (*p* < 0.001). The NAC-treated mice also showed a significant (*p* < 0.001) decrease in the serum ROS levels compared with those in the APAP-treated group.

### 2.5. Effect of TA on Serum Nitrite Levels in APAP-Treated Mice

Due to the short half-life of nitric oxide (NO) in biological fluid, serum nitrite, the stable end product of oxidation, is used to assess NO production. As shown in Figure 4C, the serum nitrite levels were significantly increased in APAP-treated mice compared to those in the control group (*p* < 0.001). However, TA pretreatment significantly reduced NO production in the APAP-treated mice (*p* < 0.05), and 5 mg/kg TA had the strongest inhibitory effect on NO production (*p* < 0.001) and was as effective as that in the NAC-treated group (*p* < 0.001).

### 2.6. Effects of TA on Serum IL-1β, IL-6, and TNF-α Levels 

Cytokines are protein mediators of inflammation and are produced following the activation of various cellular receptors. As shown in Figure 4D–F, the serum IL-1β, IL-6, and TNF-α levels were significantly increased in APAP-treated mice (*p* < 0.001). TA treatment significantly suppressed the release of these three cytokines (*p* < 0.001), which may partially diminish the inflammatory injury in the liver. The administration of 600 mg/kg NAC (positive control) also suppressed cytokine release.

### 2.7. Effects of TA on iNOS and COX-2 Expression in APAP-Treated Mouse Liver Tissues

**Figure 5:**
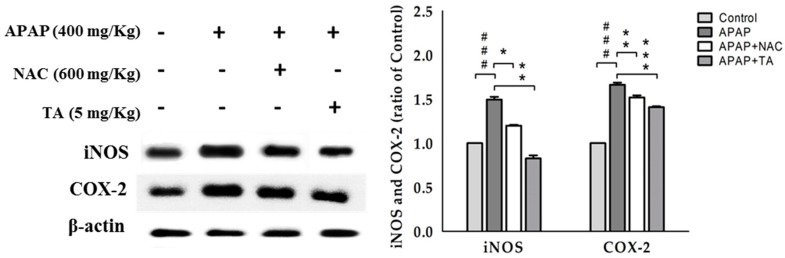
Tormentic acid inhibited iNOS and COX-2 expression in APAP-induced mice. β-actin served as a loading control. The values are reported as the means ± S.E.M. of five mice per group. ^###^ *p* < 0.01 compared with the control group; * *p* < 0.05, ** *p* < 0.01, and *** *p* < 0.001 compared with the APAP group.

We investigated TA-induced changes in iNOS and COX-2 expression in APAP-treated mice (Figure 5). The APAP treatment increased iNOS and COX-2 expression compared to that in the control. However, pretreatment with 5 mg/kg TA significantly reduced iNOS and COX-2 expression in APAP-treated mice.

### 2.8. Effect of TA on the Expression of the Antioxidant Enzymes GPx, CAT, and SOD

**Figure 6:**
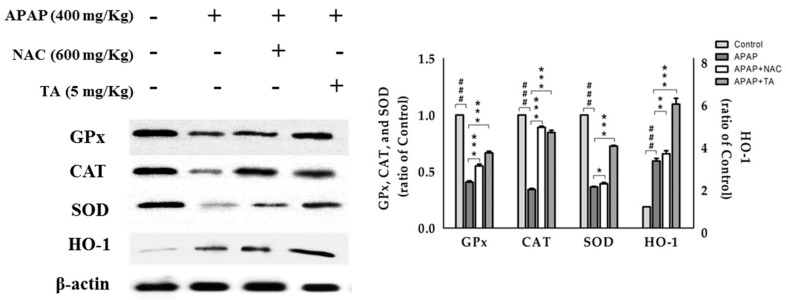
TA inhibited GPx, CAT, SOD, and HO-1 protein expression in APAP-induced mice. β-actin served as a loading control. The values are reported as the means ± S.E.M. of five mice per group. ^###^ *p* < 0.01 compared with the control group; * *p* < 0.05, ** *p* < 0.01, and *** *p* < 0.001 compared with the APAP group.

In this study, we examined changes in the protein levels of the GPx, CAT, and SOD in the liver using Western blotting analyses (Figure 6). The levels of all antioxidant enzymes were significantly decreased in the APAP-treated group compared with those in the control group, and the levels of all antioxidant enzymes were significantly maintained in the TA-treated group compared with those in the APAP-treated group.

### 2.9. Effects of TA on APAP-induced Expression of HO-1 Protein in the Mice Liver

HO-1, an antioxidant protein, is expressed to counteract various stress conditions and can ameliorate the effects of oxidative stress, inflammation, and apoptosis. The effects of TA on HO-1 expression in APAP-treated livers were analyzed by Western blotting. HO-1 levels were significantly increased in the APAP-treated group due to the APAP-induced oxidative stress. However, pretreatment with TA significantly enhanced the increase in HO-1 expression compared to that in the APAP-treated group, which suggested an increased antioxidant capacity of TA (Figure 6).

### 2.10. Effects of TA on NF-κB and IκBα in APAP-Induced Mice

**Figure 7:**
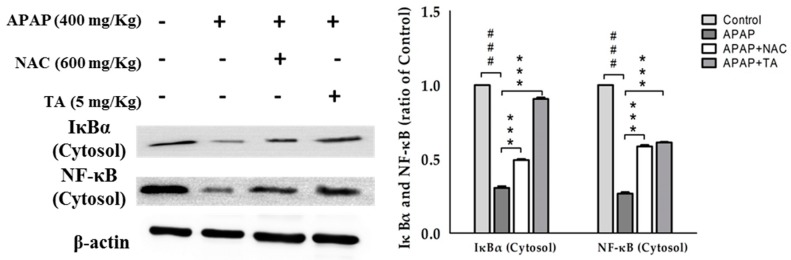
TA inhibited IκBα and NF-κB expression in APAP-induced mice. β-actin served as a loading control. The values are reported as the means ± S.E.M. of five mice per group. ^###^ *p* < 0.01 compared with the control group; *** *p* < 0.001 compared with the APAP group.

NF-κB is activated by a wide variety of pro-inflammatory cytokines, including TNF-α and IL-1β. We evaluated the cytosolic levels of NF-κB and IκBα in the liver tissues of APAP-treated mice using Western blotting analyses. As shown in Figure 7, pretreatment with TA maintained the cytosolic levels of NF-κB and IκBα, suggesting that TA may prevent the APAP-induced translocation of NF-κB into the nucleus and the degradation of IκBα.

### 2.11. Effects of TA on the APAP-Induced Phosphorylation of MAPK

**Figure 8:**
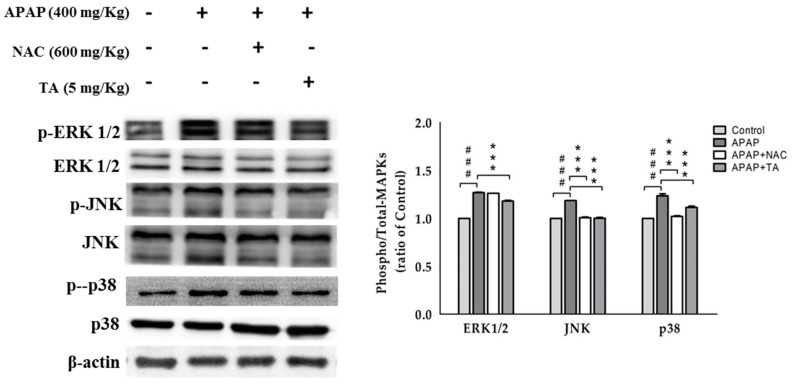
TA inhibited MAPK expression in APAP-induced mice. β-actin served as a loading control. The values are reported as the means ± S.E.M. of five mice per group. ^###^ *p* < 0.01 compared with the control group; *** *p* < 0.001 compared with the APAP group.

APAP markedly induced the phosphorylation of MAPKs, including ERK1/2, JNK, and p38 MAPK. Pretreatment with TA effectively suppressed the APAP-induced phosphorylation of all three MAPKs (Figure 8). MAPK has been reported to be involved in the NF-κB pathway and other inflammatory pathways, and our data indicated that TA could also suppress the NF-κB pathway and inflammation by inactivating MAPKs.

## 3. Discussion

An excessive dose of APAP dramatically depletes the cellular glutathione levels in the liver [34], thus accumulating oxidative stress, which can cause cell death [5]. Additionally, APAP triggers inflammation by activating resident hepatic macrophages (Kupffer cells) and the massive recruitment of circulating monocytes in mice [2]. These immune cells were believed to contribute to APAP-induced liver injury by producing pro-inflammatory cytokines and mediators, such as TNF-α and IL-1β [8]. The combination of oxidative stress accumulation and inflammatory responses affects the liver function and leads to massive hepatocyte necrosis, liver failure, or death [22,35]. 

Loquat leaf extract enriched with triterpene acids has anti-inflammatory effects by decreasing pro-inflammatory mediators and anti-oxidant effects by increasing antioxidant enzyme activities (GPx, CAT, and SOD) and HO-1, which protects against intracellular ROS [13]. Since perturbed cellular oxidative status is related to the activation of NF-κB, antioxidant activity might be another molecular mechanism underlying its anti-inflammatory effects via the inhibition of NF-κB activation [36]. TA from loquat leaf suspension cells also protects against inflammation by decreasing pro-inflammatory mediators (such as iNOS, COX-2, TNF-α) and against oxidative stress by increasing the activities of GPx, CAT, and SOD in liver tissue [27]. 

The aim of this study was to investigate the hepatoprotective mechanism of TA on APAP-induced liver injury. The present study revealed that the pretreatment of APAP-treated mice with TA significantly attenuated histological changes, including centrizonal necrosis, portal inflammation, and Kupffer cell hyperplasia (Figure 3), and the effects of TA were as beneficial as the effects of NAC, which is a potent hepatoprotective agent that increases the glutathione levels and binds to the toxic metabolites of APAP [37]. TA also prevented the elevation of liver function biomarkers, such as serum AST, ALT, and T-Bil [38], in a dose-dependent manner and inhibited abnormal lipid metabolism in TC [39] and TG due to impaired liver function following an APAP overdose [40]. These data suggest that TA effectively preserved the liver tissue integrity against APAP-induced liver injuries.

Oxidative stress is closely correlated with APAP hepatotoxicity and the elevation of ROS, and the overproduction of NO has been attributed to APAP toxicity [41]. During the formation of NAPQI by cytochrome P450, ROS is generated [6], thereby modifying bioorganic molecules and influencing various processes, such as extracellular matrix (ECM) degradation and leukocyte migration across ECM proteins [42]. These changes result in increased hepatic lipid peroxidation, in which the lipids in cell membranes are damaged [43]. 

Moreover, APAP has been shown to induce liver injury via oxidative stress through a mechanism involving reduced antioxidant enzyme levels [43]. Antioxidant enzymes such as GPx, CAT, and SOD, play critical roles in modulating the severity of APAP-induced hepatotoxicity [44]. The experimental results showed that compared with the APAP-treated group, TA treatment resulted in significant increases in the liver tissue GPx, CAT, and SOD expression.

HO-1 is an enzyme that catalyzes the rate-limiting step in the breakdown of heme to antioxidant and anti-inflammatory agents such as biliverdin, carbon monoxide, and ferrous iron. Hepatic HO-1 upregulation is rapidly triggered by several toxins, including carbon tetrachloride, endotoxin, and lipopolysaccharide/d-galactosamine [45], and provides a mechanism to counteract toxin-mediated oxidative stress. This study confirmed a significant increase in HO-1 expression following pretreatment with TA compared to that of treatment with APAP alone, suggesting that TA enhances the counteracting effect by increasing HO-1 expression beyond the normal cellular stress response against APAP-mediated oxidative stress.

NO synthesis was increased in acetaminophen toxicity [6]. Additionally, a previous report found the induction of hepatic iNOS in an acetaminophen-treated rat [46]. The physiological levels of NO chiefly contributed to the preservation of hepatic and renal hemodynamics, predominantly due to its vasodilator and antithrombogenic properties. However, Beckman et al. highlighted an important issue, as they reported that higher levels of NO may interact with superoxide anions and generate peroxynitrite [43], a potent oxidant that aggravates lipid peroxidation. Lipid peroxides and intracellular signals activate Kupffer cells, which initiate and perpetuate the inflammatory response and development of liver fibrosis [47]. In the present study, TA pretreatment ameliorated ROS generation, iNOS induction, and NO overproduction induced by an acute toxic dose of APAP in mice, thus preventing increases of lipid peroxides and TBARS, and protected cells against oxidative stress induced by APAP. 

COX-2 is believed to be the predominant cyclooxygenase involved in inflammatory responses [10]. Thus, the inhibition of COX-2 proteins might be associated with the prevention and treatment of oxidative stress-induced inflammatory diseases. The upregulation of COX-2 by APAP can be mitigated by TA, suggesting the ability of TA to suppress the inflammatory response.

Cytokines such as TNF-α, IL-1β, and IL-6 play key roles in various inflammatory processes, such as the acute-phase reaction, tissue damage, and infection [34]. Therefore, the overproduction of inflammatory cytokines is considered a prerequisite for various diseases. Based on our results, TA exerted protective effects against APAP-induced liver inflammation via the downregulation of these inflammatory molecules.

NF-κB is required for the transcription of many pro-inflammatory mediators that play important roles in acute inflammation [48]. NF-κB also modulates the expression of a variety of genes, including the genes encoding iNOS and COX-2, resulting in an increased production of TNF-α and NO in tissues [49]. APAP was shown to activate the NF-κB pathway by promoting NF-κB translocation [49] and IκBα degradation [50]. In this study, APAP-induced NF-κB translocation was blocked by either TA or NAC (Figure 7). Therefore, TA may protect against APAP-induced liver injury by sequestering NF-κB in the cytosol and inhibiting APAP-induced IκBα degradation. Additionally, TA treatment exerted a decrease of iNOS and COX-2 expression and subsequent NO production.

Three major phosphorylation reactions are observed in MAPK signaling pathways: ERK, JNK, and P38 phosphorylation [51]. Oxidative stress directly activates the JNK pathway by redox-induced alterations in JNK sequestration or the inhibition of JNK phosphatase [52]. The APAP treatment activated JNK, as reflected by the increase of JNK phosphorylation in hepatocytes in vitro and livers in vivo [53]. JNK activation is important for oxidative stress-induced hepatocyte apoptosis. The inhibition of JNK activity or suppression of the expression of the JNK gene prevented hepatic damage induced by an APAP overdose [54]. Furthermore, JNK2 has been suggested to play a beneficial role in tissue repair after an APAP overdose, and the severity of APAP-induced liver injury was recently shown to depend on JNK activation, which may be a potential therapeutic target in APAP intoxication [55]. ERK is similar to JNK in its response to oxidative stress and is associated with cellular proliferation, survival, and differentiation [53]. The ERK pathway plays an important role in APAP-induced liver injury, and its protective effects are usually accompanied by the inhibition of ERK/JNK activation [56]. The combined results of the entire study indicate that TA effectively protects against APAP-induced liver injury by preventing increases in the levels of phosphorylated ERK1/2, JNK, and P38 induced by APAP (Figure 8). 

In summary, we found that TA is a potential candidate as a natural hepatoprotective agent and the use of this selective treatment could prevent APAP-induced liver injury by suppressing ROS-mediated MAPK and NF-κB signaling pathways.

## 4. Materials and Methods 

### 4.1. Isolation of TA, Callus Induction, and Suspension Cultures 

Sterilized seeds of *E. japonica* (Department of Bio-industry and Agribusiness Administration, Taichung, Taiwan) were placed on Murashige–Skoog (MS) basal medium [57] containing 3% (*w*/*v*) sucrose and 0.3% (*w*/*v*) Gelrite. One month later, the leaves were excised (2–3 mm) from seedlings and placed on MS medium supplemented with 2.5 mg of 6-benzyladenine and 1 mg of 1-naphthalenacetic acid for callus induction. All media were adjusted to a pH of 5.8 before autoclaving for 20 min at 121 °C, and calli were cultured at 25 ± 2 °C in the dark [21]. 

The 20-day-old callus (about 3 g) induction from leaves of *E. japonica* was transferred to an Erlenmeyer flask containing 400 mL MS medium supplemented with BA, NAA, and 3% (*w*/*v*) sucrose. These cultures were incubated on a rotary shaker at 120 rpm. The temperature was maintained at 25 ± 2 °C in the dark.

The culture liquid (0.5 L) was added to a bioreactor (BioFlo 110 Fermentor, New Brunswick, NJ, USA) (4.5 L fresh culture liquid containing MS, 2.5 mg/L BA, 1 mg/L NAA and 3% sucrose) to scale up at 25 ± 2 °C for 18 days until harvest [22,58].

The suspension cells (844.5 g) were extracted with ethanol. The filtrate was concentrated under reduced pressure to yield a brown ethanol extract that was sonicated with 50% methanol and then incubated at 4 °C for 24 h to remove any water-soluble substances. The remaining substance was sonicated with 85% methanol (MeOH) at room temperature to remove insoluble substances. The filtrate was concentrated under reduced pressure to yield the white powder fraction (6.1 g) [33]. The white powder fraction (0.5 g) was assessed via chromatography on a reverse silica gel column (LiChroprep RP-18, E. Merck, 40–63 μm) using an MeOH/H_2_O gradient and then further purified using preparative high-performance liquid chromatography (PHPLC; YMC, J′Sphere series ODS-H80, 10 × 250 mm, 85% MeOH (*v*/*v*), 3 mL/min) to yield TA [33]. Nuclear magnetic resonance (NMR) spectra were measured using a Bruker spectrometer (DRX-500, Germany), as previously described [20]. 

^1^H-NMR (500 MHz, pyridine-*d*_5_): δ 1.02 (3H, s, H-25), 1.10 (3H, s, H-24), 1.13 (3H, s, H-26), 1.14 (3H, d, *J* = 6.0 Hz, H-30), 1.29 (3H, s, H-23), 1.45 (3H, s, H-29), 1.73 (3H, s, H-27), 3.07 (1H, s, H-18), 3.41 (1H, d, *J* = 9.4 Hz, H-3a), 4.13 (1H, ddd, *J* = 10.9, 9.4, 4.4 Hz, H-2b), 5.60 (1H, t, *J* = 3.1 Hz).

^13^C-NMR (125 MHz, pyridine-*d*_5_): δ 17.2 (C-30), 17.3 (C-25), 17.7 (C-26,), 18.1 (C-24), 19.5 (C-6), 24.6 (C-11), 25.2 (C-27), 26.9 (C-16), 27.4 (C-21), 27.6 (C-29), 29.7 (C-15), 29.8 (C-23), 34.0 (C-7), 39.0 (2C, C-10. C-22) 40.3 (C-4), 40.9 (C-8), 42.6 (C-14), 42.8 (C-20), 48.3 (C-9), 48.4 (C-1), 48.8 (C-17), 55.1 (C-18), 56.4 (C-5), 69.1 (C-2), 73.2 (C-19), 84.3 (C-3), 128.4 (C-12), 140.5 (C-13), 181.1 (C-28).

HR-ESI-MS *m*/*z*: 487.3429 [M – H]^−^ (calcd. for C30H47O5, 487.3418). Mass spectrometric data were generated at the Mass Spectrometry Laboratory of the National Chung Hsing University.

### 4.2. Animals and Treatments

Male ICR mice (seven to eight weeks) were obtained from the BioLASCO Taiwan Co., Ltd. (Taipei, Taiwan). The animals were housed in Plexiglas cages at a constant temperature of 22 ± 1°C and a relative humidity of 55 ± 5% on a 12 h dark-light cycle for at least two weeks before the experiment. Animals were provided food and water ad libitum. All experimental procedures were performed according to the guidelines of the Institutional Animal Ethics Committee, and the protocol was approved by the Committee for the Purpose of Control and Supervision of Experiments on Animals. 

Mice were randomly divided into the following six groups (*n* = 5 mice/group): (1) control group, (2) APAP group (negative control), (3) APAP + TA (1.25 mg/Kg) group, (4) APAP + TA (2.5 mg/Kg) group, (5) APAP + TA (5 mg/Kg) group, and (6) APAP + NAC group (positive control). In the three experimental groups, the mice were pretreated with TA (1.25, 2.5, and 5 mg/kg in 1% carboxymethyl cellulose, by intraperitoneal (i.p.)) once daily for six consecutive days. The NAC group was pretreated with NAC (600 mg/kg in phosphate buffered saline (PBS), i.p.) once daily for six days. The mice in the control and APAP groups received PBS only. One hour after the final treatment, acute liver injury was induced in all groups, except for the control group, by an i.p. injection of APAP (400 mg/kg). Fresh APAP was made immediately prior to its use in warm PBS (pH 7.4). The mice were starved for 12 h after the APAP treatment and then euthanized. Blood samples were collected from the carotid arteries. The mortality rate and body weight were recorded every day. APAP (acetaminophen) and *N*-acetylcysteine (NAC) were purchased from Sigma-Aldrich Inc. (St. Louis, MO, USA).

### 4.3. Assessment of Liver Functions

The blood was centrifuged at 1700× *g* (Beckman GS-6R, Krefeld, Germany) for 30 min at 4 °C to separate the serum. Alanine aminotransferase (ALT), aspartate aminotransferase (AST), total bilirubin (T-Bil), total cholesterol (TC), and triglyceride (TG) levels were analyzed. The biochemical parameters were analyzed using clinical test kits (HUMAN Diagnostics Worldwide, Magdeburg, Germany) with a chemical analyzer (Roche Diagnostics, Cobas Mira Plus, Rotkreuz, Switzerland), according to the manufacturer’s instructions. 

### 4.4. Histopathological Examination

Small pieces of the anterior portion of the left lateral lobe of the liver were fixed in 10 % buffered formalin and embedded in paraffin. Sections of 4–5 μm were cut and stained with hematoxylin and eosin, and then examined for histopathological changes under the microscope (Nikon, ECLIPSE, TS100, Tokyo, Japan). Images were captured with a digital camera (NIS-Elements D 2.30, SP4, Build 387) at an original magnification of 400×.

### 4.5. Hepatic Lipid Peroxidation Assays

Thiobarbituric acid reactive substances (TBARS) (in particular, malondialdehyde (MDA)), are the products of the oxidative degradation of polyunsaturated fatty acids. The MDA levels were determined to assay lipid peroxidation by measuring the absorbance at 535 nm following the reaction of MDA with thiobarbituric acid, as previously reported [59]. Briefly, 0.4 mL of liver extracts was mixed with 0.4 mL of thiobarbituric acid reagent (consisting of 0.4% thiobarbituric acid (TBA) and 0.2% butylated hydroxytoluene (BHT)). The reaction mixture was placed in a 90 °C water bath for 45 min and cooled, and an equal volume of *n*-butanol was added. The mixture was then centrifuged and the absorbance of the supernatant was recorded at 535 nm. A standard curve was obtained with a known amount of 1,1,3,3-tetraethoxypropane (TEP) using the same assay procedure. The levels of lipid peroxidation are expressed in terms of TBARS nmol/mg protein.

### 4.6. Measurement of the Levels of ROS in Serum

The effect of TA on ROS generation was determined by 2′,7′-dichlorofluorescin diacetate (DCFH-DA) (Sigma-Aldrich, St. Louis, MO, USA). Briefly, the serum was incubated with 100 μM DCFH-DA for 30 min at 37 °C in the dark place. Dichlorofluorescein (DCF) fluorescence intensities were detected by a Synergy HT Microplate Reader (BioTek Instruments) at an excitation and emission wavelength of 485 nm and 535 nm, respectively.

### 4.7. Measurement of NO/Nitrite Levels

NO production was indirectly assessed by measuring the nitrite levels in serum using a calorimetric method based on the Griess reaction [60]. Serum samples were diluted four-fold with distilled water and deproteinized by adding a 1/20 volume of zinc sulfate (300 g/L) to a final concentration of 15 g/L. After centrifugation at 10,000× *g* for 5 min at room temperature, 100 μL of the supernatant was applied to a well of a microtiter plate, followed by 100 μL of Griess reagent (1% sulfanilamide and 0.1% *N*-1-naphthylethylenediamine dihydrochloride in 2.5% polyphosphoric acid). After 10 min of color development at room temperature, the absorbance was measured at 540 nm with a Micro-Reader (Molecular Devices, Sunnyvale, CA, USA). The nitrite concentrations were determined by measuring the absorbance at 540 nm and comparing the values to a standard curve of sodium nitrite.

### 4.8. Cytokine Assays

Serum levels of TNF-α, IL-1β, and IL-6 were determined using a commercially available enzyme linked immunosorbent assay (ELISA) kit (Biosource International Inc., Camarillo, CA, USA), according to the manufacturer’s instructions. TNF-α, IL-1β, and IL-6 concentrations were determined from a standard curve.

### 4.9. Western Blot Analysis

The liver tissue was stored at −80 °C until further analysis of the protein levels. Liver tissue was homogenized in lysis buffer (0.6% NP-40, 150 mM NaCl, 10 mM HEPES (pH 7.9), 1 mM EDTA, and 0.5 mM PMSF) at 4 °C. Protein samples (50 μg) were resolved by denaturing 10% sodium dodecyl sulfate-polyacrylamide gel electrophoresis (SDS-PAGE) using standard methods and were then transferred to PVDF membranes (Immobilon, Millipore, Bedford, MA, USA) by electroblotting. The membranes were blocked with 5% skim milk and then incubated with mouse monoclonal anti-inducible nitric oxide synthase (iNOS), anti-cyclooxygenase-2 (COX-2), anti-nuclear factor-kappaB (NF-κB; p65), anti-heme oxygenase-1 (HO-1), and anti-mitogen-activated protein kinase (MAPK) antibodies, as well as the antibodies against antioxidant enzymes (superoxide dismutase (SOD), glutathione peroxidase (GPx), and catalase (CAT)) in TBS/Tween (TBST) overnight at 4 °C. The membranes were washed three times with TBST and incubated with horseradish peroxidase-conjugated secondary antibodies for 1 h at 37 °C. The membranes were washed three times before the immunoreactive proteins were detected with an enhanced chemiluminescence (ECL) reagent (Thermo Scientific, Hudson, NH, USA) and hyperfilm. The results of the Western blot analysis were quantified by measuring the relative intensity compared to the control using Kodak Molecular Imaging Software (Version 4.0.5, Eastman Kodak Company, Rochester, NY, USA) and were presented as relative intensities.

Antibodies against HO-1, an inhibitor of kappaB-alpha (IκBα), NF-κB, P38, and β-actin were obtained from Abcam (Cambridge, UK). Antibodies against iNOS, COX-2, phosphorylated extracellular regulated kinases 1/2 (p-ERK1/2), phosphorylated Jun N-terminal kinase (p-JNK), JNK, catalase (CAT), and SOD were purchased from Gene Tex (San Antonio, TX, USA). Antibodies against ERK1/2 were obtained from Cell Signaling Technology (Danvers, MA, USA). Antibodies against phosphorylated P38 (p-P38) were purchased from Millipore (Billerica, MA, USA).

### 4.10. Statistical Analysis

Data obtained from animal experiments were expressed as the means and standard errors of the means (± SEM). Statistical evaluations were conducted using one-way analysis of variance (ANOVA) followed by Scheffe’s multiple range tests. Statistical significance is expressed as * *p* < 0.05, ** *p* < 0.01, and *** *p* < 0.001.

## 5. Conclusions

TA effectively attenuated APAP-induced liver injury through various mechanisms. TA significantly suppressed APAP-induced oxidative stress. Furthermore, TA significantly decreased the levels of pro-inflammatory cytokines. In addition, the potential anti-inflammatory activity of TA, which is involved in inhibiting the MAPK and NF-κB pathways, contributes to its hepatoprotective effects. Taken together, the results indicate that TA may be a potential therapeutic strategy to prevent acute liver injury due to an acetaminophen overdose. Based on our data, TA produced from suspension cultures has considerable potential for development as a natural hepatoprotective agent for the treatment of toxic acute liver failure.

## References

[1] Tien Y.H., Chen B.H., Wang Hsu G.S., Lin W.T., Huang J.H., Lu Y.F. (2014). Hepatoprotective and anti-oxidant activities of *Glossogyne tenuifolia* against acetaminophen-induced hepatotoxicity in mice. Am. J. Chin. Med..

[2] Ju C., Tacke F. (2016). Hepatic macrophages in homeostasis and liver diseases: From pathogenesis to novel therapeutic strategies. Cell. Mol. Immunol..

[3] Ho C.M., Lee C.H., Wang J.Y., Lee P.H., Lai H.S., Hu R.H. (2014). Nationwide longitudinal analysis of acute liver failure in taiwan. Medicine (Baltimore).

[4] Dai G., He L., Chou N., Wan Y.J. (2006). Acetaminophen metabolism does not contribute to gender difference in its hepatotoxicity in mouse. Toxicol. Sci..

[5] Kumari A., Kakkar P. (2012). Lupeol prevents acetaminophen-induced in vivo hepatotoxicity by altering the Bax/Bcl-2 and oxidative stress-mediated mitochondrial signaling cascade. Life Sci..

[6] James L.P., Mayeux P.R., Hinson J.A. (2003). Acetaminophen-induced hepatotoxicity. Drug Metab. Dispos..

[7] Jaeschke H. (2003). Molecular mechanisms of hepatic ischemia-reperfusion injury and preconditioning. Am. J. Physiol. Gastrointest. Liver Physiol..

[8] Krenkel O., Mossanen J.C., Tacke F. (2014). Immune mechanisms in acetaminophen-induced acute liver failure. Hepatobiliary Surg. Nutr..

[9] James L.P., Lamps L.W., McCullough S., Hinson J.A. (2003). Interleukin 6 and hepatocyte regeneration in acetaminophen toxicity in the mouse. Biochem. Biophys. Res. Commun..

[10] Hinson J.A., Roberts D.W., James L.P. (2010). Mechanisms of acetaminophen-induced liver necrosis. Handb. Exp. Pharmacol..

[11] Zar P.P.K., Morishita A., Hashimoto F., Sakao K., Fujii M., Wada K., Hou D.X. (2014). Anti-inflammatory effects and molecular mechanisms of loquat (*Eriobotrya japonica*) tea. J. Funct. Foods.

[12] Maher K., Yassine B.A., Sofiane B. (2015). Anti-inflammatory and antioxidant properties of *Eriobotrya japonica* leaves extracts. Afr. Health Sci..

[13] Liu Y., Zhang W., Xu C., Li X. (2016). Biological activities of extracts from loquat (*Eriobotrya japonica* Lindl.): A review. Int. J. Mol. Sci..

[14] Baljinder S., Seena G., Dharmendra K., Vikas G., Bansal P. (2010). Pharmacological potential of *Eriobotrya japonica*—An overview. Int. Res. J. Pharm..

[15] Banno N., Akihisa T., Tokuda H., Yasukawa K., Taguchi Y., Akazawa H., Ukiya M., Kimura Y., Suzuki T., Nishino H. (2005). Anti-inflammatory and antitumor-promoting effects of the triterpene acids from the leaves of *Eriobotrya japonica*. Biol. Pharm. Bull..

[16] Ge J.F., Wang T.Y., Zhao B., Lv X.W., Jin Y., Peng L., Yu S.C., Li J. (2009). Anti-inflammatory effect of triterpenoic acids of *Eriobotrya japonica* (Thunb.) Lindl. Leaf on rat model of chronic bronchitis. Am. J. Chin. Med..

[17] Huang Y., Li J., Wang R., Wu Q., Li Y.H., Yu S.C., Cheng W.M., Wang Y.Y. (2007). Effect of triterpene acids of *Eriobotrya japonica* (Thunb.) Lindl. leaf on inflammatory cytokine and mediator induction from alveolar macrophages of chronic bronchitic rats. Inflamm. Res..

[18] Huang Y., Li J., Cao Q., Yu S.C., Lv X.W., Jin Y., Zhang L., Zou Y.H., Ge J.F. (2006). Anti-oxidative effect of triterpene acids of *Eriobotrya japonica* (Thunb.) Lindl. leaf in chronic bronchitis rats. Life Sci..

[19] Liu J. (2005). Oleanolic acid and ursolic acid: Research perspectives. J. Ethnopharmacol..

[20] Taniguchi S., Imayoshi Y., Kobayashi E., Takamatsu Y., Ito H., Hatano T., Sakagami H., Tokuda H., Nishino H., Sugita D. (2002). Production of bioactive triterpenes by *Eriobotrya japonica* calli. Phytochemistry.

[21] Shih C.C., Ciou J.L., Lin C.H., Wu J.B., Ho H.Y. (2013). Cell suspension culture of *Eriobotrya japonica* regulates the diabetic and hyperlipidemic signs of high-fat-fed mice. Molecules.

[22] Ho H.Y., Liang K.Y., Lin W.C., Kitanaka S., Wu J.B. (2010). Regulation and improvement of triterpene formation in plant cultured cells of *Eriobotrya japonica* Lindl. J. Biosci. Bioeng..

[23] Loizzo M.R., Bonesi M., Passalacqua N.G., Saab A., Menichini F., Tundis R. (2013). Antiproliferative activities on renal, prostate and melanoma cancer cell lines of *Sarcopoterium spinosum* aerial parts and its major constituent tormentic acid. Anticancer Agents Med. Chem..

[24] Li H.H., Su M.H., Yao D.H., Zeng B.Y., Chang Q., Wang W., Xu J. (2017). Anti-hepatocellular carcinoma activity of tormentic acid derived from suspension cells of *Eriobotrya japonica* (Thunb.) Lindl.. Plant. Cell. Tissue Organ. Cult..

[25] Rocha Gda G., Simoes M., Lucio K.A., Oliveira R.R., Coelho Kaplan M.A., Gattass C.R. (2007). Natural triterpenoids from *Cecropia lyratiloba* are cytotoxic to both sensitive and multidrug resistant leukemia cell lines. Bioorg. Med. Chem..

[26] Zhang Y., Bao F., Hu J., Liang S., Zhang Y., Du G., Zhang C., Cheng Y. (2007). Antibacterial lignans and triterpenoids from *Rostellularia procumbens*. Planta Med..

[27] Chang C.T., Huang S.S., Lin S.S., Amagaya S., Ho H.Y., Hou W.C., Shie P.H., Wu J.B., Huang G.J. (2011). Anti-inflammatory activities of tormentic acid from suspension cells of *Eriobotrya Japonica* ex vivo and in vivo. Food Chem..

[28] Banno N., Akihisa T., Tokuda H., Yasukawa K., Higashihara H., Ukiya M., Watanabe K., Kimura Y., Hasegawa J., Nishino H. (2004). Triterpene acids from the leaves of *Perilla frutescens* and their anti-inflammatory and antitumor-promoting effects. Biosci. Biotechnol. Biochem..

[29] An H.J., Kim I.T., Park H.J., Kim H.M., Choi J.H., Lee K.T. (2011). Tormentic acid, a triterpenoid saponin, isolated from *Rosa rugosa*, inhibited LPS-induced iNOS, COX-2, and TNF-alpha expression through inactivation of the nuclear factor-kappab pathway in RAW 264.7 macrophages. Int. Immunopharmacol..

[30] Zhang Q., Chang Z., Wang Q. (2006). Ursane triterpenoids inhibit atherosclerosis and xanthoma in LDL receptor knockout mice. Cardiovasc. Drugs Ther..

[31] Fogo A.S., Antonioli E., Calixto J.B., Campos A.H. (2009). Tormentic acid reduces vascular smooth muscle cell proliferation and survival. Eur. J. Pharmacol..

[32] Lin X., Zhang S., Huang R., Tan S., Liang S., Wu X., Zhuo L., Huang Q. (2014). Protective effect of tormentic acid from *Potentilla chinensis* against lipopolysaccharide/d-galactosamine induced fulminant hepatic failure in mice. Int. Immunopharmacol..

[33] Wu J.B., Kuo Y.H., Lin C.H., Ho H.Y., Shih C.C. (2014). Tormentic acid, a major component of suspension cells of *Eriobotrya japonica*, suppresses high-fat diet-induced diabetes and hyperlipidemia by glucose transporter 4 and AMP-activated protein kinase phosphorylation. J. Agric. Food Chem..

[34] Zhang Y., Zhang F., Wang K., Liu G., Yang M., Luan Y., Zhao Z. (2016). Protective effect of allyl methyl disulfide on acetaminophen-induced hepatotoxicity in mice. Chem. Biol. Interact..

[35] Cichoz-Lach H., Michalak A. (2014). Oxidative stress as a crucial factor in liver diseases. World J. Gastroenterol..

[36] Uto T., Suangkaew N., Morinaga O., Kariyazono H., Oiso S., Shoyama Y. (2010). Eriobotryae folium extract suppresses LPS-induced iNOS and COX-2 expression by inhibition of NF-kappaB and MAPK activation in murine macrophages. Am. J. Chin. Med..

[37] Lauterburg B.H., Corcoran G.B., Mitchell J.R. (1983). Mechanism of action of *N*-acetylcysteine in the protection against the hepatotoxicity of acetaminophen in rats in Vivo. J. Clin. Investig..

[38] Gao H.Y., Huang J., Wang H.Y., Du X.W., Cheng S.M., Han Y., Wang L.F., Li G.Y., Wang J.H. (2013). Protective effect of Zhuyeqing liquor, a Chinese traditional health liquor, on acute alcohol-induced liver injury in mice. J. Inflamm. (Lond.).

[39] Paul S., Islam M.A., Tanvir E.M., Ahmed R., Das S., Rumpa N.E., Hossen M.S., Parvez M., Gan S.H., Khalil M.I. (2016). Satkara (*Citrus macroptera*) fruit protects against acetaminophen-induced hepatorenal toxicity in rats. Evid. Based Complement. Altern. Med..

[40] Chen C., Krausz K.W., Shah Y.M., Idle J.R., Gonzalez F.J. (2009). Serum metabolomics reveals irreversible inhibition of fatty acid beta-oxidation through the suppression of PPARalpha activation as a contributing mechanism of acetaminophen-induced hepatotoxicity. Chem. Res. Toxicol..

[41] El-Shafey M.M., Abd-Allah G.M., Mohamadin A.M., Harisa G.I., Mariee A.D. (2015). Quercetin protects against acetaminophen-induced hepatorenal toxicity by reducing reactive oxygen and nitrogen species. Pathophysiology.

[42] Coito A.J. (2011). Leukocyte transmigration across endothelial and extracellular matrix protein barriers in liver ischemia/reperfusion injury. Curr. Opin. Organ. Transplant..

[43] Beckman J.S., Beckman T.W., Chen J., Marshall P.A., Freeman B.A. (1990). Apparent hydroxyl radical production by peroxynitrite: Implications for endothelial injury from nitric oxide and superoxide. Proc. Natl. Acad. Sci. USA.

[44] Michael Brown J., Ball J.G., Wright M.S., Van Meter S., Valentovic M.A. (2012). Novel protective mechanisms for S-adenosyl-L-methionine against acetaminophen hepatotoxicity: Improvement of key antioxidant enzymatic function. Toxicol. Lett..

[45] Kim S.J., Kim K.M., Park J., Kwak J.H., Kim Y.S., Lee S.M. (2013). Geniposidic acid protects against d-galactosamine and lipopolysaccharide-induced hepatic failure in mice. J. Ethnopharmacol..

[46] Gardner C.R., Heck D.E., Yang C.S., Thomas P.E., Zhang X.J., DeGeorge G.L., Laskin J.D., Laskin D.L. (1998). Role of nitric oxide in acetaminophen-induced hepatotoxicity in the rat. Hepatology.

[47] Contreras-Zentella M.L., Hernandez-Munoz R. (2016). Is liver enzyme release really associated with cell necrosis induced by oxidant stress?. Oxid. Med. Cell. Longev..

[48] Carayol N., Chen J., Yang F., Jin T., Jin L., States D., Wang C.Y. (2006). A dominant function of IKK/NF-kappaB signaling in global lipopolysaccharide-induced gene expression. J. Biol. Chem..

[49] Li Y.Y., Huang S.S., Lee M.M., Deng J.S., Huang G.J. (2015). Anti-inflammatory activities of cardamonin from *Alpinia katsumadai* through heme oxygenase-1 induction and inhibition of NF-kappaB and MAPK signaling pathway in the carrageenan-induced paw edema. Int. Immunopharmacol..

[50] Guo Q., Shen Z., Yu H., Lu G., Yu Y., Liu X., Zheng P. (2016). Carnosic acid protects against acetaminophen-induced hepatotoxicity by potentiating Nrf2-mediated antioxidant capacity in mice. Korean J. Physiol. Pharmacol..

[51] Yiang G.T., Yu Y.L., Lin K.T., Chen J.N., Chang W.J., Wei C.W. (2015). Acetaminophen induces JNK/p38 signaling and activates the caspase-9–3-dependent cell death pathway in human mesenchymal stem cells. Int. J. Mol. Med..

[52] Bourdi M., Korrapati M.C., Chakraborty M., Yee S.B., Pohl L.R. (2008). Protective role of c-Jun *N*-terminal kinase 2 in acetaminophen-induced liver injury. Biochem. Biophys. Res. Commun..

[53] Conde de la Rosa L., Schoemaker M.H., Vrenken T.E., Buist-Homan M., Havinga R., Jansen P.L., Moshage H. (2006). Superoxide anions and hydrogen peroxide induce hepatocyte death by different mechanisms: Involvement of JNK and ERK MAP kinases. J. Hepatol..

[54] Hanawa N., Shinohara M., Saberi B., Gaarde W.A., Han D., Kaplowitz N. (2008). Role of JNK translocation to mitochondria leading to inhibition of mitochondria bioenergetics in acetaminophen-induced liver injury. J. Biol. Chem..

[55] Du K., Williams C.D., McGill M.R., Jaeschke H. (2014). Lower susceptibility of female mice to acetaminophen hepatotoxicity: Role of mitochondrial glutathione, oxidant stress and c-jun N-terminal kinase. Toxicol. Appl. Pharmacol..

[56] Noh J.R., Kim Y.H., Hwang J.H., Gang G.T., Kim K.S., Lee I.K., Yun B.S., Lee C.H. (2013). Davallialactone protects against acetaminophen overdose-induced liver injuries in mice. Food Chem. Toxicol..

[57] Murashige T., Skoog F. (1962). A revised medium for rapid growth and bio assays with tobacco tissue cultures. Physiol. Plant..

[58] Ho H.Y., Lin W.C., Kitanaka S., Chang C.T., Wu J.B. (2008). Analysis of bioactive triterpenes in *Eriobotrya japonica* Lindl. by high-performance liquid chromatography. J. Food Drug Anal..

[59] Huang G.J., Deng J.S., Huang S.S., Lee C.Y., Hou W.C., Wang S.Y., Sung P.J., Kuo Y.H. (2013). Hepatoprotective effects of eburicoic acid and dehydroeburicoic acid from *Antrodia camphorata* in a mouse model of acute hepatic injury. Food Chem..

[60] Huang G.J., Deng J.S., Huang S.S., Shao Y.Y., Chen C.C., Kuo Y.H. (2012). Protective effect of antrosterol from *Antrodia camphorata* submerged whole broth against carbon tetrachloride-induced acute liver injury in mice. Food Chem..

